# Big-data-driven modeling unveils country-wide drivers of endemic schistosomiasis

**DOI:** 10.1038/s41598-017-00493-1

**Published:** 2017-03-28

**Authors:** Lorenzo Mari, Marino Gatto, Manuela Ciddio, Elhadji D. Dia, Susanne H. Sokolow, Giulio A. De Leo, Renato Casagrandi

**Affiliations:** 1 Politecnico di Milano, Dipartimento di Elettronica, Informazione e Bioingegneria, Milano, IT 20133 Italy; 2 Ministère de la Santé et de l’Action Sociale, Dakar, BP 4024 Senegal; 3 Stanford University, Hopkins Marine Station, Pacific Grove, CA 93950 USA; 4 University of California, Marine Science Institute, Santa Barbara, CA 93106 USA

## Abstract

Schistosomiasis is a parasitic infection that is widespread in sub-Saharan Africa, where it represents a major health problem. We study the drivers of its geographical distribution in Senegal via a spatially explicit network model accounting for epidemiological dynamics driven by local socioeconomic and environmental conditions, and human mobility. The model is parameterized by tapping several available geodatabases and a large dataset of mobile phone traces. It reliably reproduces the observed spatial patterns of regional schistosomiasis prevalence throughout the country, provided that spatial heterogeneity and human mobility are suitably accounted for. Specifically, a fine-grained description of the socioeconomic and environmental heterogeneities involved in local disease transmission is crucial to capturing the spatial variability of disease prevalence, while the inclusion of human mobility significantly improves the explanatory power of the model. Concerning human movement, we find that moderate mobility may reduce disease prevalence, whereas either high or low mobility may result in increased prevalence of infection. The effects of control strategies based on exposure and contamination reduction via improved access to safe water or educational campaigns are also analyzed. To our knowledge, this represents the first application of an integrative schistosomiasis transmission model at a whole-country scale.

## Introduction

Schistosomiasis is a major parasitic infection that affects about 250 million individuals in many areas of the developing world and that puts at risk about 700 million people in regions where the disease is endemic^1^. It is a major cause of mortality, being directly responsible for the death of about 12,000 people yearly^2^ and a co-factor in at least 200,000 deaths annually^3^. Schistosomiasis is also an important determinant of morbidity, with 20 million people suffering severe consequences from the disease^4^ and an estimated burden of 4.5 million disability-adjusted life years^5^. These figures make schistosomiasis the second most common parasitic disease after malaria and likely the deadliest among neglected tropical diseases. Schistosomiasis is disproportionately concentrated in sub-Saharan Africa, which accounts for at least 90% of cases worldwide^1^.

**Figure 1:**
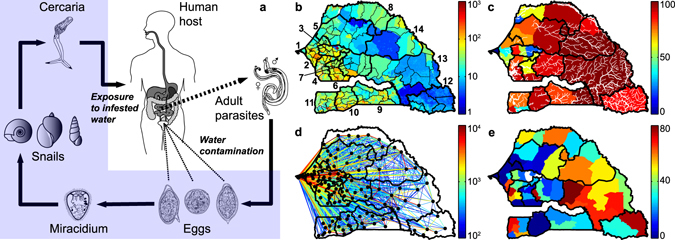
Schistosomiasis transmission cycle (**a**) and data for model application (**b**–**e**). (**a**) Paired adult worms within human hosts produce eggs (left to right: *S*. *mansoni*, *S*. *japonicum*, *S*. *haematobium*) that are shed through feces or urine and hatch into miracidia. Miracidia infect species-specific intermediate snail hosts (left to right: genus *Biomphalaria*, *Bulinus*, *Oncomelania*), which then shed free-swimming cercariae that can penetrate human skin and eventually develop into reproductive schistosomes. (**b**) High-resolution population density map of Senegal [inhabitants km^−2^]. Black lines indicate administrative boundaries (thick/thin lines are for regions/arrondissements). Regions are numbered as follows: 1–Dakar, 2–Thiès, 3–Diourbel, 4–Fatick, 5–Louga, 6–Kaolack, 7–Kaffrine, 8–Saint-Louis, 9–Kolda, 10–Sédhiou, 11–Ziguinchor, 12–Kédougou, 13–Tambacounda, 14–Matam. (**c**) People living in rural settings [%] (colors) and rivers of Senegal (thick/thin white lines are for perennial/ephemeral rivers). (**d**) Human mobility fluxes in year 2013 [number of people] estimated from anonymous mobile phone traces; the flux between any two arrondissements (say *i* and *j*, $i\ne j$) is obtained as *K*_*i*_ *Q*_*ij*_ (see Table 1). Only fluxes ≥100 people are displayed as links between arrondissement-level population centroids. (**e**) Prevalence of urogenital schistosomiasis [% of infected people] according to the national surveys operated by the Senegalese Ministry of Health. Data are shown at the scale of health districts and cover the timespan 1996–2013. See SI for details on data sources. The drawings in panel a are from the Centers for Disease Control and Prevention (CDC, Parasites: Schistosomiasis, http://www.cdc.gov/parasites/schistosomiasis/biology.html; last date of access: 03/02/2017). The maps in panels b–e have been created with QGIS 2.4 (QGIS Development Team, QGIS: A free and open source geographic information system, http://www.qgis.org/; last date of access: 03/02/2017) and MATLAB R2015b (MathWorks, MATLAB, http://www.mathworks.com/products/matlab/; last date of access: 03/02/2017).

The disease is caused by trematode parasites belonging to the genus *Schistosoma* ^6^. Most human infections are caused by three species, namely *S*. *haematobium*, *S*. *mansoni* or *S*. *japonicum*. These parasites need as obligate intermediate hosts some species of freshwater snails belonging to the genus *Bulinus* (for *S*. *haematobium*), *Biomphalaria* (for *S*. *mansoni*) or *Oncomelania* (for *S*. *japonicum*). The geographical distribution of schistosomes is thus linked to the species-specific range of the snail host habitat. The infectious form of the parasite for humans is a freely swimming, short-lived larval stage, known as cercaria, that is shed by infected snails. Cercariae can infect humans exposed to infested water by penetrating their skin. Within the human body, they develop into sexually mature adult parasites that live for years, mating and producing hundreds to thousands of eggs daily. Eggs leave the human host through urine (*S*. *haematobium*) or feces (*S*. *mansoni* or *S*. *japonicum*). After reaching freshwater, they hatch into so-called miracidia, a second short-lived larval form of the parasite that can infect snails. Miracidia replicate asexually in snails, which then daily shed hundreds of cercariae into water, thus completing the parasite's life cycle. The population dynamics of human and snail hosts, as well as of different vital stages of the parasite, are thus essential components in the description of the transmission cycle of schistosomiasis (Fig. 1a).

Spatial coupling mechanisms are very important for the spread, persistence and infection intensity of schistosomiasis^7, 8^. Parasites may in fact be carried in advective flows along canals and streams as larvae, moved between aquatic and riparian habitats inside snail hosts, or transported by human hosts as adult worms. While larval transport and snail movement may represent significant propagation pathways for the disease only over short spatial scales (e.g. in the order of hundreds of meters^9, 10^) or long temporal windows (e.g. because of habitat expansion following water resources development^11, 12^), human mobility can play a significant role in disease propagation within and from endemic areas^13–15^. People can in fact be exposed to water infested with cercariae while visiting endemic regions and import the parasites back to their home communities; also, if infected, they can contribute to water contamination while traveling outside their home communities. Both mechanisms are expected to favor parasite dispersion and may even introduce schistosomes into villages that were previously disease-free.

Human mobility differs from the ecohydrological pathways of schistosomiasis propagation in that human movement can occur between otherwise environmentally unconnected areas, over larger spatial scales, and over shorter (and less predictable) temporal scales^16^. As a matter of fact, despite recent advances in the modeling of human mobility^17–19^, there still remain fundamental limits to our understanding of where, when, why and how people move^20, 21^. Standard mobility models have been found to perform poorly in the African context^22^. Therefore, proxies of human mobility that can be remotely acquired, properly anonymized and quantitatively elaborated represent an invaluable tool to inform epidemiological modeling. In this respect, the analysis of Call Detail Records (CDRs) from mobile phone users represents one of the most promising tools to infer human movement patterns^23, 24^ – also in an epidemiological context, as shown by the rapidly increasing number of studies that make use of CDRs to characterize human mobility^25–37^.

In this work we explore country-wide patterns of schistosomiasis transmission in Senegal, where the urogenital form of the infection is widespread^38, 39^. Schistosomiasis represents a major health problem in the country, being the third disease (after malaria and lymphatic filariasis) in terms of years lived with disability^40^. We apply a spatially explicit network model of schistosomiasis accounting for the dynamics of human hosts, intermediate snail hosts and larval parasite stages (*Methods* and Fig. S1). Because of the large spatial scale of interest, the exposure/contamination rates for the human host communities are assumed to be spatially heterogeneous to account for local differences in transmission risk. For the same reason, human mobility is here retained as the most important mechanism for the spatial spread of the disease; human movement patterns are extracted from a large dataset of anonymized CDRs (more than 15 billion records) made available by Sonatel, the largest Senegalese telecommunication provider (with a customer base of more than 9 million people). The model is parameterized with georeferenced data on population abundance, socioeconomic conditions and freshwater distribution, and calibrated against the most up-to-date regional estimates of urogenital schistosomiasis prevalence currently available at the Senegalese Ministry of Health (Figs 1b–e and S2). Specific aim of this work is to assess the impact of environmental conditions and human mobility on schistosomiasis transmission from local to regional scales. To that end, we compare the performances of four model set-ups characterized by different assumptions regarding the spatial grain of environmental heterogeneity and the inclusion of human mobility (*Methods*). We also illustrate how the analysis of local heterogeneities in disease transmission can help guide resource allocation in the fight against the disease, with the overarching goal of showcasing how mathematical modeling can be used for societal development, namely by assisting decision makers in the fight against a poverty-reinforcing infection like schistosomiasis.

## Results

### Drivers of endemic schistosomiasis in Senegal

**Figure 2:**
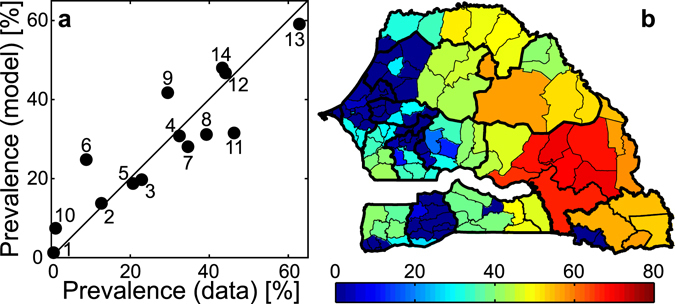
Reference model simulation and comparison with epidemiological evidence. (**a**) Quantitative agreement between simulated disease prevalence at the regional scale and the available data (labels as in Fig. 1b) for the best-fit model accounting for fine-grained spatial heterogeneity in transmission risk and human mobility estimated from CDRs (M4, reference model). (**b**) Projected schistosomiasis prevalence [% of people infected] at the scale of third-level administrative units as obtained from the reference model. Calibrated parameter values: *β*_0_ = 5.5 · 10^−3^ [days^−1^], *χ*_0_ = 2.2 · 10^−6^ [days^−1^ parasites^−1^], $\varphi =4.3\cdot {10}^{-1}$, $\xi =1.1\cdot {10}^{2}$. See Table 1 for parameter definitions and *Methods* for details on the model. The map in panel (b) has been created with QGIS 2.4 (QGIS Development Team, QGIS: A free and open source geographic information system, http://www.qgis.org/; last date of access: 03/02/2017) and MATLAB R2015b (MathWorks, MATLAB, http://www.mathworks.com/products/matlab/; last date of access: 03/02/2017).

Of the four tested model set-ups (*Methods*), the one accounting for both a fine-grained description of spatially heterogeneous transmission risk and human mobility (M4) performs best in reproducing regional schistosomiasis prevalence values (Fig. S3). The simulation results from this model are in good quantitative agreement with the available epidemiological data (Fig. 2a, coefficient of determination data vs. model *R* ^2^ = 0.76). The average absolute data-model deviation is 6.0%, while the largest differences are found for the regions of Kaolack (6), Ziguinchor (11) and Kolda (9), where the model overestimates (Kaolack and Kolda) or underestimates (Ziguinchor) disease prevalence by more than 10%. Conversely, a model accounting for fine-grained environmental heterogeneity but neglecting human mobility (M3) shows worse explanatory power at the regional scale (*R* ^2^ = 0.64), while models characterized by coarse-grained heterogeneity, either accounting for (M2) or neglecting (M1) human mobility, cannot even capture the spatial variability of regional prevalence (*R* ^2^ < 0, see Supplementary Information, SI). The best-fit set-up including fine-grained spatial heterogeneity and human mobility (M4) is thus retained as reference model and used for further analyses. Although calibrated with regional prevalence data, the reference model can project infection patterns throughout the country at the spatial scale of third-level administrative units (so-called arrondissements, Fig. 2b).

### Spatial patterns of infection intensity

From an epidemiological standpoint, the reference model projects a country-wide schistosomiasis prevalence of about 21%, with a regional maximum in Tambacounda (59% of clinically infected people). Sensitivity analysis (Fig. S4) suggests that these figures are quite robust to (moderate) changes in model parameterization, with the baseline human exposure rate, the schistosome mortality rate and the threshold for clinical infection in humans being responsible for the largest variations in model predictions. The model also projects an Average Parasite Burden (APB, see *Methods*) of approximately 7.2 parasites per person, with arrondissement-level values ranging between 2.3 and 12.7 (Fig. S5a). The frequency distribution of APB is actually bimodal, with marked peaks around 3 and 10 parasites per person (Fig. S5b). The within-host parasite distributions in each of the 123 arrondissements can be well approximated by negative binomial distributions (Fig. S5c), with aggregation parameters ranging from ≈24.5 in arrondissements where APB is lowest to ≈20.5 where APB is highest, and dispersion indexes ranging between ≈1.1 and ≈1.5 for increasing values of APB (Fig. S5d).

### Role of human mobility

**Figure 3:**
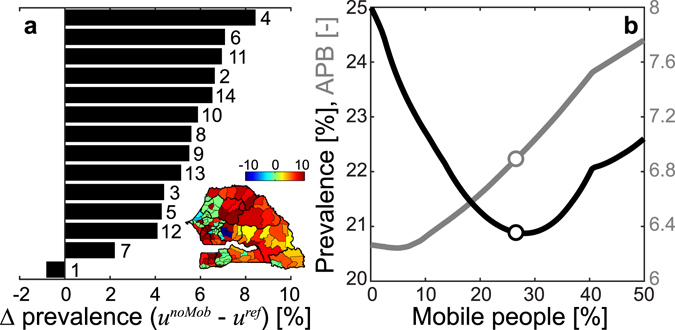
Effects of human mobility on schistosomiasis prevalence. (**a**) Differences [%] in regional disease prevalence (labels as in Fig. 1b) as predicted by the reference model (M4, mobility matrix estimated from CDRs) or by a model with the same parameter values but no mobility (mobility matrix set to be the identity matrix); positive values indicate higher prevalence in the model without mobility (absolute differences sorted in decreasing order). Inset: arrondissement-scale differences in infection prevalence: positive values indicate again higher prevalence in the model without mobility. (**b**) Projected country-scale disease prevalence (black, left axis) and APB (gray, right axis) as a function of the fraction of mobile people (those who leave their home arrondissement at least once a year). The dots indicate infection prevalence and APB corresponding to the mobility level inferred from CDR analysis (26%). Different levels of human mobility have been obtained by artificially manipulating the mobility matrix estimated from data (SI). Parameters as in Fig. 2. The map in the inset of panel (a) has been created with QGIS 2.4 (QGIS Development Team, QGIS: A free and open source geographic information system, http://www.qgis.org/; last date of access: 03/02/2017) and MATLAB R2015b (MathWorks, MATLAB, http://www.mathworks.com/products/matlab/; last date of access: 03/02/2017).

Contrasting the reference model against a simulation with the same parameter values but no mobility (SI) is useful to enucleate the impact of human mobility on the spatial patterns of schistosomiasis prevalence. Regional disease prevalence is found to be higher in all regions but Dakar if human mobility is completely switched off (Fig. 3a). Instead, the impact of human mobility at the arrondissement level is relatively more diversified in space: the effects of the mobility switch-off are less pronounced in the westernmost part of the country, where smaller increments (or even decrements in some arrondissements) of disease prevalence are predicted in the absence of mobility (inset of Fig. 3a). More in general, it is possible to contrast the reference model with simulations in which the human mobility rate is artificially manipulated, i.e. decreased or increased with respect to the country-wide estimate from CDR analysis (26% of mobile people in a one-year interval); this also requires a suitable redistribution of mobility fluxes (SI). According to model projections, country-scale APB shows an increasing trend with increasing mobility, while disease prevalence attains a well-defined minimum for intermediate levels of mobility – remarkably, close to the actual estimate of mobility obtained from CDRs (Fig. 3b). Therefore, changes in mobility can alter within-host parasite distributions at the community level in a way that nontrivially influences disease prevalence.

### Disease control

**Figure 4:**
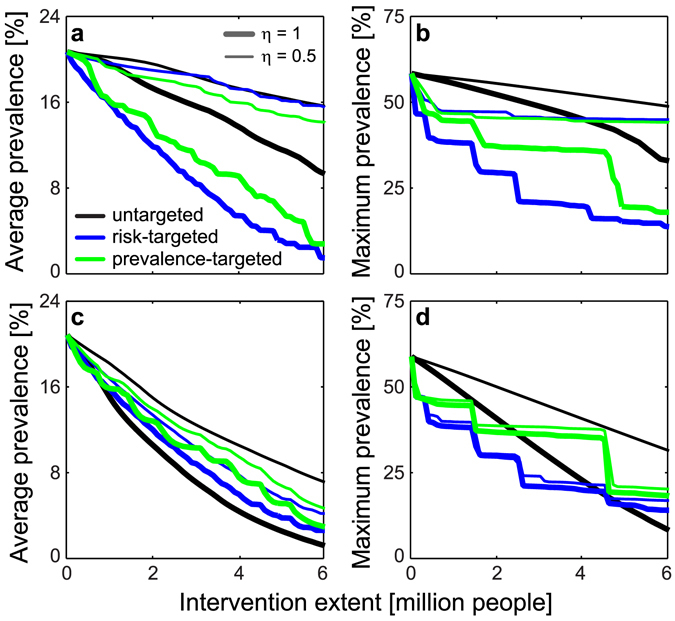
Evaluation of large-scale control strategies. (**a**) Effect of WASH interventions on country-wide average schistosomiasis prevalence. (**b**) As in panel a, but for maximum regional prevalence. (**c**,**d**) As in panels a,b, but for IEC campaigns. Targeted actions prioritize communities where transmission risk (as quantified by the quantity *ρ*_*i*_ *ω*_*i*_, see Table 1 and *Methods*; blue lines) or schistosomiasis prevalence (green) is highest. Results are shown for two different values of the expected efficiency (*η*) of the control actions. See SI for details on WASH/IEC interventions. Parameters as in Fig. 2.

The reference model is finally used to evaluate the effects of so-called WAter, Sanitation and Hygiene (WASH) or Information, Education and Communication (IEC) strategies aimed to reduce the burden of schistosomiasis in Senegal through prevention of human exposure and contamination (SI). Concerning WASH (Fig. 4a,b), the model suggests that targeted interventions, prioritizing either high-risk communities (where schistosomiasis transmission is expected to be highest because of the synergistic effect of rural living conditions and abundance of freshwater environments) or high-prevalence communities, may be more effective than untargeted ones in reducing both the average and the maximum regional prevalence of infection. Specifically, risk-targeted actions are predicted to be the most effective option if the expected efficiency of the interventions is high; conversely, prevalence-targeted interventions may be more effective in reducing average disease prevalence for low expected efficiency. Different results are obtained with IEC campaigns (Fig. 4c,d). In this case, untargeted actions may represent the most effective option to reduce average prevalence, namely if the expected efficiency of the interventions is high and the plan involves at least ≈1 million people (≈5 millions for maximum regional prevalence). In case of smaller-scale plans or low expected efficiency, targeted actions are predicted again to be more efficient; in this case, the effects of prioritizing high-risk vs. high-prevalence communities depend on the planned extent of the intervention.

## Discussion

In this work we have proposed a big-data-driven modeling framework to study the country-scale dynamics of schistosomiasis transmission in Senegal. We have shown that a fine-grained description of the socioeconomic and environmental heterogeneities involved in local disease transmission is crucial to capturing the spatial variability of the regional prevalence patterns, and that the inclusion of human mobility estimates obtained from mobile phone traces significantly improves the explanatory power of the model. In this respect, the best-fit model including these two drivers of disease transmission is able to quite reliably reproduce large-scale patterns of schistosomiasis prevalence throughout the country. To our knowledge, this represents the first application of a country-scale schistosomiasis transmission model making an integrative use of socioeconomic, environmental and mobility proxies to infer human exposure/contamination patterns.

Human mobility can have remarkable implications in the definition of the country-scale prevalence patterns of an endemic disease like schistosomiasis. Specifically, our results show that the level of human mobility estimated from CDRs may be associated with lower values of schistosomiasis prevalence compared to those obtained in a no-mobility scenario at regional and country scales. This (possibly quite unexpected) finding can be explained by the fact that, under the current mobility scenario, the largest mobility fluxes are attracted by the most populated and urbanized regions (Dakar, Thiès and Diourbel), where schistosomiasis transmission is low. The higher disease prevalence predicted in the absence of mobility in prevalently rural areas is a clear indication of the importance of mobility directed to urban areas in preventing local disease transmission. Conversely, mobility may represent a risk factor for people living in prevalently urban areas, as found for the region of Dakar and several other areas in the western part of the country, where local infection prevalence is expected to be higher in the presence of mobility, clearly because of movement to/from rural areas where schistosomiasis thrives. Numerical simulations also show that, should propensity to moving increase with respect to what currently inferred from CDRs, country-wide average schistosomiasis prevalence could become higher as well. Country-wide APB as a function of mobility shows a different trend, namely a nearly monotonic increase for higher levels of mobility. This result seems to agree with other modeling studies that have reported a positive relationship between human mobility and transmission emergence, parasite burden and disease spread^7^. *Per se*, however, APB may represent a relatively poor epidemiological indicator for schistosomiasis dynamics. By accounting for the stratification of the infection^41^, instead, our framework shows that human mobility may play a more complex role in the definition of the spatial patterns of schistosomiasis prevalence than previously thought, especially at small spatial scales.

In this respect, one possible limitation of our model is the current choice of third-level administrative entities as computational units. Such choice is the result of trading off between data availability (the spatial resolution of country-wide data sets and sources is often relatively coarse) and computational cost (which clearly increases with the granularity of the model) on the one hand, and the need of preserving at least some of the heterogeneity that is inherent^42, 43^ to schistosomiasis transmission (as resulting, for instance, from regional differences in water availability and living conditions, or from human mobility) on the other. Because all variability within third-level administrative units is averaged out, our model misses some possibly important sources of heterogeneity (such as the demographic structure of the human population and small-scale changes in environmental conditions). For this reason, our description of schistosomiasis dynamics can be seen as a first-order approximation of the actual mechanisms responsible for the transmission of this disease. The current spatial granularity of the model may also result in an underestimation of the aggregation of community-level parasite distributions. The best-fit model suggests relatively low parasite aggregation throughout the country (arrondissement-level parasite distributions characterized by aggregation parameters >20 and dispersion indexes close to 1), which however is also a typical feature of models based on worm burden stratification^44^ (see also SI). We argue that high-resolution models targeted to specific regions of the country, where more accurate data – including snail distribution and infection status – may also be available^37^, could better elucidate the actual role of spatial heterogeneities and coupling mechanisms at fine spatiotemporal scales, and possibly help identify the focal hotspots of disease transmission.

High-resolution models, in turn, would call for a more-in-depth look at several sources of complexity that have been neglected at this stage. In particular, human exposure and contamination are directly related to water contact patterns, which are linked to demography and social structure. Including a simple, yet realistic, demographic model able to describe the age structure of the population at risk, as well as to track intra- and inter-annual changes in local population abundance, could greatly improve the reliability of epidemiological projections^44^. While census microdata could be used to describe long-term mobility trends^45^, CDRs can be exploited to derive (or validate) short-term movement patterns and/or time-varying mobility fluxes. A closer look at the connectivity matrices derived from CDRs shows in fact that human movement is highly heterogeneous, not only in space but also in time (Fig. S6). Overall mobility displays clear weekly patterns (especially in the most urbanized regions, such as Dakar), longer-term trends (possibly linked to seasonal economic activities, such as agriculture and fishing) and sudden pulses^46^. Religious gatherings attract pilgrims from all regions of Senegal and thus produce remarkable mobility fluxes, with the temporary displacement of hundreds of thousands of people, like in the case of the Grand Magal de Touba and Kazu Rajab (also held in Touba). Mobility pulses of this kind have been both anecdotally^47^ and quantitatively^35^ associated with cholera outbreaks, yet their possible role in the transmission of endemic diseases like schistosomiasis is still to be investigated.

From a biological perspective, a detailed description of parasite-host interactions and in-host parasite biology^48^, as well as of the ecology of the obligate intermediate snail host of schistosomes^49, 50^ has yet to be integrated in our modeling framework. As the presence of snail hosts is a major determinant of transmission risk, accounting for the spatiotemporal variability of the environmental drivers (most notably, water temperature and rainfall^51^) that influence their distribution and abundance could greatly improve the explanatory power of the model. Particular attention should be devoted to studying the possible interplay between the seasonality of environmental signals (which *per se* can induce complex dynamics^52^) and time-varying human mobility, which could introduce non-trivial effects on disease transmission^53^. Integrating the ecology of the intermediate host into the modeling framework described here is also deemed crucial to planning and optimizing non-conventional intervention strategies based on biological snail control. A promising avenue is represented by the restoration, possibly via village-based aquaculture^54^, of a native prawn species (*Macrobrachium vollenhovenii*) that has virtually disappeared from the Senegal river because of anthropogenic alterations, namely the construction of the Diama dam in the 1980's. *M*. *vollenhovenii* is a voracious snail predator, whose feeding activity can permanently interrupt disease transmission by suppressing the intermediate host population^55, 56^.

When building a fine-scale account of the ecological interactions that are relevant to schistosomiasis transmission, hydrological dispersal of the snail intermediate hosts, as well as of the larval stages of the parasite, has also to be included^7, 16, 37^. A detailed description of hydrological connectivity at a fine spatial scale may also allow studying the effects of agricultural development^57^, which requires the implementation of irrigation schemes and the construction of dam reservoirs. These interventions, in turn, can induce severe perturbations of the natural matrix that influences the population dynamics of snails and their natural enemies^11, 58^. As an example, the development of irrigation channels following the construction of the Diama dam resulted in increased transmission of *S*. *haematobium* and the introduction of *S*. *mansoni* in villages upriver of the dam, with a globally unprecedented velocity of transmission^59^. These observations highlight the importance of addressing the conflict between the need for water resources development and infectious disease management^8, 11^.

Current measures for fighting schistosomiasis are principally focused on chemotherapy^3, 6^. Senegal has implemented a national control program (*Programme National de Lutte contre la Bilharziose*, PNLB) since 1999^38^. The PNLB is still ongoing, but the scarcity of large-scale data on treatment coverage and intervention effectiveness prevented us from including the effects of any mass drug administration in our country-wide model. Because chemotherapy does not confer permanent immunity, preventing infection by improving access to safe water and spreading awareness about disease transmission can represent a sustainable path towards reducing the burden of schistosomiasis. In this respect, our analysis of control strategies based on water contact conforms with a meta-analysis of observational studies^60^ that found that both safe water supplies and adequate sanitation are associated with significantly lower odds of schistosomiasis infection. However, safe water supplies may reduce contact with environmental water, but cannot completely avert it; similarly, sanitation can prevent snail infection, but the availability of adequate sanitation does not guarantee its use^61^. Our analysis also indicates that disease control efforts should be guided by a thorough understanding of the drivers that determine local heterogeneities in transmission risk, especially if resource availability is limited (a commonplace in the fight of neglected tropical diseases in developing countries) and/or a high efficiency of the interventions cannot be taken for granted. It is also to be remarked that no single action based on WASH or IEC alone would realistically be able to stop schistosomiasis transmission in Senegal. Sensitivity analysis seems to suggest that a combination of exposure prevention and chemotherapy could prove effective for large-scale disease control in humans. All these observations stress the importance of a comprehensive approach to schistosomiasis management^62^.

The results of this study encourage us to further elaborate on the current modeling framework to create operational tools aimed at supporting decision makers in the design of effective plans for disease control, as well as at informing citizens about the geography of transmission risk at different spatial scales. These decision-support systems should be able to accommodate real-time data assimilation (epidemiological reports, ecological surveys, demographic updates), in addition to reliable projections of relevant environmental drivers, like temperature and rainfall^63, 64^. Achieving greater detail in the description of epidemiological dynamics, human mobility, ecological interactions, water resources development and control plans is unlikely to be feasible at the country scale, but should be possible if looking at smaller spatial scales (e.g. specific regions of Senegal), at which the underlying modeling hypotheses can be substantiated by knowledge gathered *in situ*, possibly with the help of local institutions. The lessons learned from local experiences could then be scaled up to define country-scale strategies to help the fight against schistosomiasis transmission in Senegal, which could thus serve as an example for other countries (and/or other diseases) in sub-Saharan Africa.

## Methods

### A spatially explicit network model for schistosomiasis dynamics

The human population is subdivided into *n* communities (following e.g. administrative boundaries, health zones or geographical divides). Within each community *i*, the resident human population (of size *K*_*i*_) is considered to be ‘stratified’^41, 44, 48^, i.e. divided into different infection classes characterized by increasing parasite burden *p* (from *p* = 0 to some maximum abundance *p* = *P*). Except for this, the population is assumed to be well-mixed within each community (no demographic/socioeconomic grouping). Let ${H}_{i}^{p}$ be the abundance of individuals in community *i* who host exactly *p* parasites. Furthermore, let *S*_*i*_ and *I*_*i*_ be the densities of susceptible and infected snails in community *i*, and let *C*_*i*_ and *M*_*i*_ be the concentrations of cercariae and miracidia in the freshwater resources accessible to community *i*. Following the transmission cycle of schistosomiasis (Fig. 1), disease transmission can be described by the following set of *n*(*P* + 5) differential equations:

$$\begin{array}{lll}{\dot{H}}_{i}^{0} & = & {\mu }_{H}({K}_{i}-{H}_{i}^{0})-{ {\mathcal F} }_{i}{H}_{i}^{0}+{\gamma }^{1}{H}_{i}^{1}\\ {\dot{H}}_{i}^{p} & = & { {\mathcal F} }_{i}{H}_{i}^{p-1}-({\mu }_{H}+{\alpha }_{H}^{p}+{ {\mathcal F} }_{i}+{\gamma }^{p}){H}_{i}^{p}+{\gamma }^{p+1}{H}_{i}^{p+1}\quad \mathrm{(0} < p < P)\\ {\dot{H}}_{i}^{P} & = & { {\mathcal F} }_{i}{H}_{i}^{P-1}-({\mu }_{H}+{\alpha }_{H}^{P}+{\gamma }^{P}){H}_{i}^{P}\\ {\dot{S}}_{i} & = & {\mu }_{S}({N}_{i}-{S}_{i})-b{M}_{i}{S}_{i}\\ {\dot{I}}_{i} & = & b{M}_{i}{S}_{i}-({\mu }_{S}+{\alpha }_{S}){I}_{i}\\ {\dot{C}}_{i} & = & {\pi }_{C}{I}_{i}-{\mu }_{C}{C}_{i}\\ {\dot{M}}_{i} & = & {{\mathscr{G}}}_{i}-{\mu }_{M}{M}_{i}\mathrm{.}\end{array}$$

The first *n*(*P* + 1) equations of the model describe the dynamics of human hosts, the following 2*n* the dynamics of intermediate snail hosts, the last 2*n* the dynamics of the larval stages of the parasite. Model variables and parameters are summarized in Table 1, while a graphical representation of the transmission model is provided in Fig. S1.

**Table 1:** Model variables and parameters.

Symbol	Variable
${H}_{i}^{p}$	abundance of people hosting *p* parasites in community *i*
*S*_*i*_	abundance of susceptible snails in community *i*
*I*_*i*_	abundance of infected snails in freshwater used by community *i*
*C*_*i*_	abundance of cercariae in freshwater used by community *i*
*M*_*i*_	abundance of miracidia in freshwater used by community *i*
*μ*_*H*_	baseline human mortality rate
*K*_*i*_	human population size in community *i*
${ {\mathcal F} }_{i}=a{\sum }_{j=1}^{n}{Q}_{ij}{\theta }_{j}{C}_{j}$	force of infection for people in community *i*
*a*	probability of schistosome establishment in human hosts
**Q** = [*Q*_*ij*_]	human mobility matrix (between communities *i* and *j*)
*θ*_*i*_	rate of human exposure to cercariae in community *i*
*γ* ^*p*^ = *pμ*_*P*_	parasite resolution rate for human hosts with *p* parasites
*μ*_*P*_	schistosome mortality rate
${\alpha }_{H}^{p}=p{\alpha }_{H}$	schistosomiasis-related mortality rate for human hosts with *p* parasites
*α*_*H*_	additional human mortality rate induced by each parasite
*P*	maximum number of parasites in human hosts
*T*	parasite threshold for clinical infection in humans
*μ*_*S*_	baseline snail mortality rate
*N*_*i*_	snail population size in community *i*
*b*	rate of snail exposure to miracidia
*α*_*S*_	infection-related mortality rate in snails
*π*_*C*_	cercarial shedding rate by infected snails
*μ*_*C*_	mortality rate of cercariae
${{\mathscr{G}}}_{i}={\pi }_{M}{\delta }_{i}{\sum }_{j=1}^{n}{Q}_{ji}{{\mathscr{W}}}_{j}\mathrm{/2}$	rate of freshwater contamination by infected people in community *i*
*π*_*M*_	miracidial shedding rate by infected humans
*δ*_*i*_	probability of freshwater contamination by infected people in community *i*
${{\mathscr{W}}}_{i}={\sum }_{p=1}^{P}p{H}_{i}^{p}$	abundance of schistosomes carried by residents of community *j*
*μ*_*M*_	mortality rate of miracidia
${\beta }_{i}=a\frac{{\pi }_{C}}{{\mu }_{C}}{\theta }_{i}{N}_{i}={\beta }_{0}(1+\varphi {\rho }_{i}{\omega }_{i})$	synthetic human exposure rate
${\chi }_{i}=\frac{b}{2}\frac{{\pi }_{M}}{{\mu }_{M}}{\delta }_{i}={\chi }_{0}(1+\xi {\rho }_{i}{\omega }_{i})$	synthetic human contamination rate
*ρ*_*i*_	rurality score of community *i*
*ω*_*i*_	freshwater availability score of community *i*
*β*_0_	baseline value of the synthetic human exposure rate (calibrated)
*χ*_0_	baseline value of the synthetic human contamination rate (calibrated)
*ϕ*	shape parameter for the human exposure rate (calibrated)
*ξ*	shape parameter for the human contamination rate (calibrated)

The top and middle parts of the table summarize the state variables and the parameters of the schistosomiasis transmission model described in the *Methods* section, while the bottom part describes the synthetic human exposure and contamination rates obtained after introducing equilibrium assumptions for larval abundances and rescaling the remaining state variables (see SI for details).

As for the dynamics of human hosts, *μ*_*H*_ is the baseline per capita mortality rate, while *μ*_*H*_ *K*_*i*_ represents the total birth rate, here assumed to be constant (i.e. leading to a constant community size *K*_*i*_ in the absence of disease-induced mortality). Human hosts progress from one infection class to the following because of exposure to water infested with cercariae. Specifically, ${ {\mathcal F} }_{i}=a{\sum }_{j=1}^{n}{Q}_{ij}{\theta }_{j}{C}_{j}$ is the force of infection for the inhabitants of community *i*: **Q** = [*Q*_*ij*_] is a row-stochastic matrix (i.e. a matrix in which rows sum to one) that describes the probability that residents of community *i* travel to community *j* (possibly different from their home community as a result of human mobility), *θ*_*j*_ is the human exposure rate, i.e. the rate at which human hosts either permanently or temporarily staying in community *j* are exposed to contaminated freshwater (exposure rate is assumed to be possibly community-dependent, so as to account for the geographical heterogeneity of living conditions), and *a* is the probability that a cercaria successfully develops into a reproductive adult parasite following contact with a human host. The term *γ* ^*p*^ represents the parasite resolution rate, i.e. the transition rate from infection class *p* to infection class *p* − 1 because of the death of one parasite (*γ* ^*p*^ = *pμ*_*P*_, with *μ*_*P*_ being the per capita schistosome mortality rate). Disease-related mortality in humans is accounted for by the term ${\alpha }_{H}^{p}$, which describes increasing mortality for increasing parasite burden (${\alpha }_{H}^{p}=p{\alpha }_{H}$, where *α*_*H*_ is the additional mortality rate possibly experienced by an infected host because of the presence of each adult parasite). As for the dynamics of snail hosts, *μ*_*S*_ is the baseline mortality rate, whereas *μ*_*S*_ *N*_*i*_ is the constant recruitment rate (local population size in the absence of the parasite is *N*_*i*_). The parameter *b* represents the exposure rate of susceptible snails to miracidia in the freshwater environment. Exposure triggers a transition to the infected compartment (possible delays between exposure and onset of infectivity^52^ are neglected here for the sake of model minimality). Infective snails suffer from an extra-mortality rate *α*_*S*_. As for the dynamics of larval stages, cercariae are shed by infected snails at rate *π*_*C*_ and die at rate *μ*_*C*_. Similarly, miracidia are shed by infected human hosts and die at rate *μ*_*M*_; specifically, the total human contamination rate for community *i* is ${{\mathscr{G}}}_{i}={\pi }_{M}{\delta }_{i}{\sum }_{j=1}^{n}{Q}_{ji}{{\mathscr{W}}}_{j}\mathrm{/2}$, with *π*_*M*_ being the shedding rate of miracidia by infected humans, *δ*_*i*_ the possibly site-specific probability of contaminating accessible freshwater, and *Q*_*ji*_ the probability that inhabitants of community *j* come in contact with freshwater in community *i*. Shedding is assumed to be proportional to the total number ${{\mathscr{W}}}_{j}\mathrm{/2}$ of adult parasite pairs undergoing sexual reproduction in the human hosts of community *j*, with ${{\mathscr{W}}}_{j}={\sum }_{p=1}^{P}p{H}_{j}^{p}$. Note that this may represent an overestimate, especially at low parasite counts^48^.

From the model, it is straightforward to evaluate disease prevalence (namely, by assuming that a minimum number *T* of parasites per host is required for the infection to be clinically apparent), the APB (a common measure of community-level infection intensity) and suitable indicators of parasite aggregation within each community, such as the dispersion index (defined as the ratio between the sample variance of the parasite distribution and the APB) and the aggregation parameter (obtained by fitting a negative binomial to the simulated parasite distribution). These quantities can be evaluated *ex*-*post*, i.e. as outputs of model simulations. Technical details are reported in SI.

### Model set-ups

We use four model set-ups (M1–M4) to investigate the role played by spatial heterogeneity and human mobility in schistosomiasis transmission. The different set-ups are characterized by either a coarse-grained (M1 and M2) or a fine-grained (M3 and M4) description of environmental heterogeneity, and by either neglecting (M1 and M3) or taking into account (M2 and M4) human mobility. As for spatial heterogeneity, communities are grouped into two clusters according to transmission risk (either low or high) in M1 and M2, while they are endowed with site-specific exposure/contamination rates (depending on environmental and socioeconomic factors) in M3 and M4. As for human movement, the mobility matrix is set to be the identity matrix in M1 and M3, while its entries are estimated from CDRs in M2 and M4; note that in M1 and M3 the system describing schistosomiasis transmission reduces to a set of spatially disconnected local models. Technical details on the evaluation of environmental heterogeneity and human mobility from georeferenced data are given in SI.

### Application of the model to Senegal

The model is run at the spatial scale of the arrondissements (third-level administrative units as of 2013). A high-resolution population density map (Fig. 1b) is used to obtain local values of *K*_*i*_ (Fig. S2a). To simplify the structure of the model some equilibrium assumptions are made for the larval stages of the parasite. As a result, the synthetic exposure (*β*_*i*_, accounting also for snail abundance) and contamination (*χ*_*i*_) rates are introduced. These parameters are assumed to increase with the product (Fig. S2b,c) between the fraction of people living in rural areas (*ρ*_*i*_) and the availability of environmental freshwater (*ω*_*i*_, measured as the total length of the rivers encompassed in each spatial unit, Fig. 1c), i.e. ${\beta }_{i}={\beta }_{0}\mathrm{(1}+\varphi {\rho }_{i}{\omega }_{i})$ and ${\chi }_{i}={\chi }_{0}\mathrm{(1}+\xi {\rho }_{i}{\omega }_{i})$. Human mobility is estimated from the anonymized movement routes of about 9 million Sonatel mobile phone users (corresponding to more than 60% of the Senegalese population) collected for one year, from January 1 to December 31, 2013. The entries of the mobility matrix **Q** are assumed to be proportional to the number of phone calls made by users living in site *i* while being in site *j* (Fig. 1d). The home site of each user is identified as the place where the most calls are made during night hours (7 pm–7 am). The model is calibrated against regional estimates of urogenital schistosomiasis prevalence (Fig. S2d) upscaled from the health-district data available at the Senegalese Ministry of Health (Fig. 1e). The prevalence of schistosomiasis in the country is periodically evaluated during national surveys conducted within the PNLB. Model calibration is performed against the data that are currently in use at the Ministry of Health, and that refer to surveys conducted through standard diagnostic techniques (urine testing via reagent strips, followed by filtration and microscopic examination of samples positive for haematuria) in schools selected from all of the 14 regions of Senegal between 1996 and 2013. Performing model calibration at the regional (rather than a finer) spatial scale is deemed to decrease the effects of the uncertainties possibly associated with census and/or epidemiological data. Details and references for model parameterization and calibration are reported in SI, along with a description of some control strategies aimed at decreasing disease burden by preventing human exposure and contamination. Although used here to study schistosomiasis transmission in Senegal, our modeling framework can easily be applied to other geographical regions, provided that suitable data for model calibration are available.

## Electronic supplementary material

Supplementary information
