# Supplementary material for: Big-data-driven modeling unveils country-wide drivers of endemic schistosomiasis
Source: Sci Rep. 2017 Mar 28;7:489. doi: 10.1038/s41598-017-00493-1 (PMC5428445; doi:10.1038/s41598-017-00493-1)
Supplement: Supplementary file 1 — Supplementary information [file 41598_2017_493_MOESM1_ESM.pdf]

Supplementary Information

Big-data-driven modeling unveils country-wide drivers of endemic schistosomiasis

Lorenzo Mari[1,⋆], Marino Gatto[1], Manuela Ciddio[1], Elhadji D. Dia[2], Susanne H. Sokolow[3,4], Giulio A. De Leo[3], and Renato Casagrandi[1,†]

[1]Politecnico di Milano, Dipartimento di Elettronica, Informazione e Bioingegneria, Milano, IT 20133, Italy

[2]Ministère de la Santé et de l'Action Sociale, Dakar, BP 4024, Senegal

[3]Stanford University, Hopkins Marine Station, Pacific Grove, CA 93950, USA

[4]University of California, Marine Science Institute, Santa Barbara, CA 93106, USA

[⋆]lorenzo.mari@polimi.it

[†]renato.casagrandi@polimi.it

ABSTRACT

In this supplementary file we provide additional information on the application of the schistosomiasis tranmission model to the Senegal case study, including details on the evaluation of environmental heterogeneity. Moreover, we describe in full details the elaborations performed on the mobile phone data and the related human mobility matrices, and discuss how control strategies based on exposure and contamination reduction can be implemented in the model. Six additional figures are also included.

1 Details on model implementation

Administrative boundaries and population distribution

The territory of Senegal is currently divided into first-level administrative units (14 regions), each subdivided into second-level units (45 departments overall), each of which is further divided into third-level units (123 arrondissements overall, as of 2013; a revision is currently underway). In order to apply the model to study large-scale patterns of schistosomiasis dynamics in Senegal, human communities are identified with arrondissements. Note that arrondissements can actually include several human settlements/villages, yet we refrain from choosing smaller units for the sake of computational feasibility and data availability.

Population size for each arrondissement (K_i, Fig. S2a) is obtained from a high-resolution population distribution map (Fig. 1b in the main text) available from the AfriPop project, which is part of the WorldPop project (data available online at GeoData Institute, University of Southampton, WorldPop: High resolution age-structured population distribution maps, http://www.worldpop.org.uk/; last last date of access: 03/02/2017). Data include 2014 estimates of population distribution with a spatial resolution of 30 arcsec (approx 100 m at the equator), and national totals adjusted to match United Nations estimates. The total number of people living in each arrondissement is thus computed by summing the 2014 population estimates of the grid squares that fall within the relevant administrative boundaries. Population-weighted centroids are also evaluated for each third-level administrative unit (see again Fig. S2a).

Simplifying hypotheses for model application

By noting that the lifespan of the larval stages of the parasite is much shorter than those of the other biological agents involved in the transmission cycle of the disease (up to a few days vs. months/years[1]), the concentrations of cercariae and miracidia can be considered at their equilibrium values (as obtained by setting $\dot{C}_i = 0$ and $\dot{M}_i = 0$). By also rescaling the state variables as

$$h_i^p = \frac{H_i^p}{K_i}\,, \quad s_i = \frac{S_i}{N_i}\,, \quad y_i = \frac{I_i}{N_i}\,,$$

the model described in the *Methods* section of the main text can be written as

$$\begin{aligned}
\dot{h}_i^0 &= \mu_H(1-h_i^0) - F_i h_i^0 + \gamma^1 h_i^1 \\
\dot{h}_i^p &= F_i h_i^{p-1} - (\mu_H + \alpha_H^p + F_i + \gamma^p) h_i^p + \gamma^{p+1} h_i^{p+1} \qquad (0 < p < P) \\
\dot{h}_i^P &= F_i h_i^{P-1} - (\mu_H + \alpha_H^P + \gamma^P) h_i^P \\
\dot{s}_i &= \mu_S(1-s_i) - G_i s_i \\
\dot{y}_i &= G_i s_i - (\mu_S + \alpha_S) y_i\,,
\end{aligned}$$

with

$$F_i = \sum_{j=1}^{n} Q_{ij}\beta_j y_j\,, \quad \beta_j = a\frac{\pi_C}{\mu_C}\theta_j N_j\,, \quad G_i = \chi_i \sum_{j=1}^{n} Q_{ji} W_j\,, \quad \chi_i = \frac{b}{2}\frac{\pi_M}{\mu_M}\delta_i\,, \quad W_j = K_j \sum_{p=1}^{P} p h_j^p\,.$$

The parameters β_i and χ_i represent, respectively, aggregated exposure and contamination rates – the former of which also accounts for the local abundance of the intermediate snail hosts. Although simplified, this formulation can indeed ease the

application of the model to real case studies for which detailed information on the heterogeneity of transmission risk might not be readily available.

Spatial heterogenity of human exposure and contamination

The use of the model outlined above requires the specification of the parameters β_i and χ_i, i.e. of the (possibly) site-specific exposure and contamination rates. Both parameters are clearly related to rurality and availability of environmental freshwater. Rural communities that lack access to piped drinking water and improved sanitation, and that have to resort to unsafe water sources for their primary needs, are in fact both more prone and more conducive to schistosomiasis transmission[2,3]. Conversely, the availability of adequate water provisioning and sanitation infrastructures may represent an effective protection against schistosomiasis, as shown in a recent systematic review of available field data[4]. However, neither can safe water supplies completely avert human contact with environmental freshwater, especially in water-rich regions, nor can the presence of adequate sanitation guarantee *per se* its use[5]. Therefore, the causal pathway through which water and sanitation affect disease transmission still remains elusive – even more so in developing countries where understanding exposure and mechanisms of spread would be most important[6]. Moreover, in the simplified formulation of the epidemiological model described above, exposure risk also depends on local snail population abundances, which are also certainly influenced by rurality and environmental freshwater availability. Malacological surveys suggest that occurrence of the snail species involved in schistosomiasis transmission is widespread in Senegal[7]. However, the lack of quantitative data on an appropriate spatial scale precludes the use of these observations to inform the model about the spatial distribution of snail populations. Habitat suitability for freshwater snails can be mapped via geostatistical methods[8,9], which however require a considerable amount of field observations, currently not available for Senegal.

To parameterize the model, we assume that the synthetic exposure and contamination rates β_i and χ_i increase with the fraction of people living in rural areas, ρ_i, and the availability of environmental freshwater, ω_i; more specifically, β_i and χ_i are assumed to increase with the product $\rho_i \omega_i$, which may represent a simple (yet comprehensive) proxy for transmission risk heterogeneity linked to socioeconomic and environmental conditions. The rurality index ρ_i is available at the department level through the Global Atlas of Helminth Infections (Fig.1c, data available online at GAHI, Maps and data, http://www.thiswormyworld.org; last last date of access: 03/02/2017), based on mapping and spatial analysis of cross-sectional survey data[10]. The freshwater abundance index ω_i is computed by summing up the length of perennial and ephemeral rivers encompassed in each department (see again Fig. 1c in the main text; data available from DIVA-GIS Development Team, DIVA-GIS, http://www.diva-gis.org/gdata; last last date of access: 03/02/2017); the resulting distribution is then normalized by its maximum value, so that values of ω_i are distributed in a 0–1 interval, and spatially downscaled [upscaled] by assigning department data to the relevant arrondissements [regions]. The spatial distribution of the product $\rho_i \omega_i$ that is actually assumed to drive the spatial distribution of the aggregated exposure and contamination rates is shown in Fig. S2b.

The four model set-ups tested in this work (see *Methods* in the main text) are characterized by two different levels of spatial granularity for the environmental heterogeneity informing transmission risk. Two sets of communities, endowed with either low or high transmission risk, are identified for the set-ups with coarse-grained spatial heterogeneity (M1 and M2). Risk groups are formed via k-means clustering ($k = 2$) of the arrondissement-level values of $\rho_i \omega_i$ (Fig. S2c). Exposure and contamination rates in low-risk [high-risk] communities are respectively set to β_{low} and χ_{low} [β_{high} and χ_{high}]. Conversely, linear relationships

between arrondissement-level values of $\rho_i \omega_i$ and the transmission parameters are established for the set-ups with fine-grained spatial heterogeneity (M3 and M4), i.e.

$$\beta_i = \beta_0 (1 + \phi \rho_i \omega_i)\,, \quad \chi_i = \chi_0 (1 + \xi \rho_i \omega_i)\,,$$

where

$$\beta_0 = a \frac{\pi_C}{\mu_C} \theta_0 N_0\,, \quad \chi_0 = \frac{b}{2} \frac{\pi_M}{\mu_M} \theta_0'$$

are the baseline values of the synthetic exposure and contamination rates, respectively, while ϕ and ξ are two coefficient accounting for the combined effects of rurality and freshwater abundance on exposure and contamination[11].

Model simulation and evaluation of epidemiological indicators

As schistosomiasis is endemic in Senegal, model outputs are evaluated by running the epidemiological model up to convergence to steady state starting from an initial condition in which human communities are set to be completely uninfected ($h_i^0 = 1$ and $h_i^p = 0$ for $0 < p \leq P$ in all arrondissements), while the prevalence of infected snails is initially set to 5% ($s_i = 0.95$ and $y_i = 0.05$ in all arrondissements). Note that the model produces an estimate of the distribution of human hosts among infection classes (and of the prevalence of susceptible/infected snails in each arrondissement). Comparing this output with prevalence data requires the definition of an infection threshold. According to commonly accepted epidemiological evidence[17], a minimum number of parasites within a human host is in fact required for pathogen reproduction to be effective and to lead to a positive result during clinical screening. The infection threshold (T) thus represents the minimum parasite burden above which human hosts are considered to be infected. The prevalence u_i^M of clinically infected human hosts in each arrondissement can be evaluated from the model as the sum of the prevalences of the infection classes characterized by parasite burden larger than T, i.e.

$$u_i^M = \frac{\sum_{p=T+1}^{P} h_i^p}{\sum_{q=0}^{P} h_i^q}\,.$$

Infection prevalence can be upscaled to departmental/regional scales via weighted averaging (i.e. using arrondissement population sizes as weights).

The model also allows to easily estimate the Average Parasite Burden (APB), a standard measure that is routinely used in epidemiology to quantify the community-level intensity of infection, in addition to disease prevalence, and that closely relates to morbidity. APB can be defined as the mean number of parasites hosted in each resident of a human community (say i), i.e.

$$\text{APB}_i = \frac{\sum_{p=1}^{P} p H_i^p}{\sum_{p=0}^{P} H_i^p}\,.$$

Therefore, in our framework, evaluation of APB is done *ex-post* – that is, APB can be seen as an output (rather than as a state variable) of the model. Although APB represents the basis for the traditional approach to describe schistosomiasis transmission dynamics[18], it forcedly neglects the heterogeneity that typically characterizes the distribution of schistosomes among human hosts within a community. Models based on a Stratified Worm Burden (SWB) approach[17], like the one used in this work, can

be used to effectively overcome this limitation of purely APB-based models. SWB models have also been shown to better reproduce the epidemiological dynamics actually observed in the field[19,20].

In the SWB approach, the analysis of APB can be usefully complemented with the evaluation of some indicators of the heterogeneity of parasite distribution, such as the aggregation parameter k_i, obtained by fitting a negative binomial distribution to the simulated parasite loads in each community i, or the dispersion index D_i, defined as the ratio between the sample variance of parasite distribution (σ_i^2) and the sample APB$_i$ within each community. A typical feature of SWB-based models parameterized with realistic values of the mortality rates of human hosts and adult schistosomes is that they tend to produce parasite distributions with relatively low aggregation. In fact, by using a simple SWB model applied to an isolated and stationary host population subject to a constant force of infection, it has been shown[19] that the equilibrium distribution of parasite burden within human hosts is controlled by the ratio between the human and the schistosome mortality rates. Specifically, in absence of human demographic turnover ($mu_H = 0$ and $\alpha_H^p = 0$) the distribution is strictly Poisson, well approximated by a negative binomial otherwise. The highly aggregated distributions typically observed in egg-count data can be produced by more complex SWB models accounting for a detailed description of in-host biology (including e.g. parasite mating, aggregation, density-dependent fecundity and random egg-release)[20].

Model calibration

Some of the model parameters can be reliably estimated from the literature or from epidemiological/demographic records. Specifically, the baseline mortality rates of human hosts, snails and parasites can be evaluated as the inverse of the average lifetimes of people in Senegal (61 years[21], hence $\mu_H = 4.5 \cdot 10^{-5}$ days^{-1}), snail intermediate hosts (about 1 year[22], hence $\mu_S = 2.7 \cdot 10^{-3}$ days^{-1}) and schistosomes (about 5 years[22], hence $\mu_P = 5.5 \cdot 10^{-4}$ days^{-1}), respectively. From these figures we have $\mu_H / \mu_P = 0.082$. Following a field study conducted in an endemic area of Sudan[23], parasite-induced mortality in human hosts is set to $\alpha_H = 1.1 \cdot 10^{-7}$ days^{-1} parasite^{-1}. The extra-mortality suffered by infected snails is set to $\alpha_S = 1.4 \cdot 10^{-2}$ days^{-1}, according to the observation that the lifespan of infected snails is about two months[22]. As for parasite load in human hosts, we consider a maximum burden of $P = 150$ parasites and a threshold for infection $T = 10$ parasites[17]. The human population of each community is thus divided into $P+1$ classes, with classes $T < p \leq P$ being considered as infected.

Conversely, numerical fitting is necessary to calibrate the parameters describing human exposure and contamination risk. Here we use the urogenital schistosomiasis prevalence data collected by the Senegalese Ministry of Health during the first national survey carried out in 1996 and periodically updated since then (namely, in 2003, 2009, 2010, 2012 and 2013) in the context of the *Programme National de Lutte contre la Bilharziose* operated by the Senegalese Ministry of Health since 1999[7]. Epidemiological information is available at the health-district level (Fig. 1e in the main text). Raw data were collected among children in selected schools from all 14 regions of Senegal through standard diagnostic techniques (for urogenital schistosomiasis: urine testing via reagent strips, followed by filtration and microscopic examination of samples positive for haematuria). Despite relative small sample sizes (as an example, 5,000 children from 100 schools in 20 districts were tested in the most recent campaign), which may lead to some uncertainty at fine spatial scales, this dataset is currently in use at the Senegalese Ministry of Health as the most reliable and up-to-date picture of the spatial distribution of the disease in the country.

Calibration parameters vary among the four model set-ups: specifically, the parameters that need be calibrated are $\{\beta_{low}, \chi_{low}, \beta_{high}, \chi_{high}\}$ or $\{\beta_0, \chi_0, \phi, \xi\}$, respectively for the set-ups with coarse-grained (M1 and M2) or fine-grained (M3

and M4) environmental heterogeneity. Note that no parameters involving human mobility are calibrated in any of the model set-ups (see below). Calibration is performed independently for each model set-up by minimizing the residual sum of squares of reported (u_r^D) vs. modeled (u_r^M) values of schistosomiasis prevalence in each region r (Fig. S2d), i.e.

$$RSS = \sum_r (u_r^D - u_r^M)^2 \, .$$

Regional infection prevalences can be readily upscaled from the epidemiological dataset at the health-district level via weighted averaging (i.e. using health-district population sizes as weights; each health-district value is assigned to the region where the health-district lies). Numerical fitting is performed with the Nelder-Mead simplex method[24].

Because all the model set-ups have the same number of calibration parameters, performance comparison can simply be performed by evaluating the coefficient of determination

$$R^2 = 1 - \frac{RSS}{TSS} \, ,$$

where

$$TSS = \sum_r \left(u_r^D - \langle u_r^D \rangle\right)^2$$

is the total sum of squares and $\langle u_r^D \rangle$ is the mean of the regional prevalence values. Note that R^2 can also be negative, namely if $RSS > TSS$.

2 Human mobility

Inference of human mobility from mobile phone traces

Mobility is estimated from the anonymized movement routes of Sonatel mobile phone users collected for one year, from January 1 to December 31, 2013. Sonatel is the main telecommunications provider of Senegal, with more than 9 million subscribers in the country. The 2013 mobile phone dataset contains more than 15 billion CDRs. Each record includes an anomynous identifier for the user making the call, as well as information about when (time stamp) and where (antenna tower) the call was initiated. Matrix $\mathbf{Q} = [Q_{ij}]$ is defined in this work as the probability that people usually living in community (arrondissement) i come in contact with freshwater in community (arrondissement) j ($j = 1 \dots 123$, including i). We assume that this probability is proportional to the time spent in arrondissement j, and that the number of phone calls made by a user while being in arrondissement j is also proportional to the time spent in that arrondissement. Therefore, the entries Q_{ij} of matrix $\mathbf{Q}$ are assumed to be proportional to the number of phone calls made by users usually living in arrondissement i while being in arrondissement j.

To characterize human mobility patterns, first we upscale antenna-level data to the spatial scale of arrondissements, namely by assigning the traces originated at each antenna to the relevant arrondissement based on the geographical position of the antenna. Then, we use CDRs to identify the ‘home’ (residential) arrondissement for each anonymous user. Following a definition often used in the context of CDR analysis and related epidemiological applications[12], we define home as the site (arrondissement) where most calls are made by a user during night hours (from 7pm to 7am) over the whole dataset (i.e. over a

timespan of one year). If several arrondissements match this criterion, home is randomly selected among the arrondissements that host most of the night calls made by the user. Afterwards, for each arrondissement i, the number of calls made in arrondissement j by users whose home site has been identified with i is extracted from the dataset. This number, properly divided by the total number of calls made by users usually living in arrondissement i (independently of the location where the call originates from), represents an estimate of the entries of the mobility matrix (graphically shown in Fig. 1d in the main text). The mobility matrix $\mathbf{Q}$ so derived is used in the model set-ups where human movement is accounted for (M2 and M4), and can be made available upon request for replication purposes.

A useful indicator of mobility is the community-level fraction of the population of each arrondissement that leaves its home site at least once during the time window of interest (e.g. one year in Fig. 1d in the main text), i.e. $m_i = 1 - Q_{ii}$. Yearly values of m_i estimated from CDRs fall in the range 0.15–0.44 for the different arrondissements, with a country-wide weighted average

$$\langle m \rangle = \frac{\sum_i m_i K_i}{\sum_i K_i} = 0.26\,.$$

Artificial manipulation of human mobility patterns

To elucidate the role of human movement on schistosomiasis transmission, it may be useful to analyze some scenarios in which mobility is different from CDR-based estimates. To that end, the mobility matrix estimated from CDRs has to be artificially manipulated. Given an average country-wide mobility $\langle m^* \rangle$, the local values of m_i^* can be obtained by redistributing the total number $\langle m^* \rangle \sum_i K_i$ of mobile people proportionally to the contribution $m_i K_i$ of each arrondissement i to the total number $\langle m \rangle \sum_i K_i$ of mobile people in the reference case, so that

$$m_i^* = \frac{\langle m^* \rangle}{\langle m \rangle} m_i\,.$$

The diagonal elements Q_{ii}^* of the modified mobility matrix $\mathbf{Q}^*$ can thus be obtained as $Q_{ii}^* = 1 - m_i^*$, while the off-diagonal entries Q_{ij}^* are assumed to be proportional to the contribution of each destination site j to overall mobility from site i as estimated from CDRs, i.e.

$$Q_{ij}^* = \frac{\langle m^* \rangle}{\langle m \rangle} Q_{ij}\,.$$

Matrix $\mathbf{Q}^*$ so obtained can then be fed to the model set-ups originally calibrated with $\mathbf{Q}$ estimated from CDRs (set-ups M2 and M4). Clearly, $\langle m^* \rangle = 0$ (or, equivalently, $\mathbf{Q}^* = \mathbf{I}$, with $\mathbf{I}$ being the identity matrix) corresponds to a no-mobility scenario (set-ups M1 and M3).

As a word of caution, we acknowledge that changes in human mobility could actually produce effects on the resulting mobility matrix that are far more complex than the simple manipulations described above. In that case, human mobility models – such as the so-called gravity[13] or radiation[14] models, which can be calibrated against movement patterns extracted from CDRs[15] – could be used to better analyze different mobility scenarios. Note, however, that recent studies have shown that standard mobility models may perform poorly in the sub-Saharan context, thus warranting extra-care in their use[16].

3 The fight against schistosomiasis in Senegal: analysis of intervention strategies based on exposure/contamination reduction

WAter, Sanitation and Hygiene (WASH) interventions

The effect of WASH actions aimed at increasing access to safe drinking water and improved sanitation[3] can be seen as equivalent, in our simplified formulation of the spatially heterogeneous exposure and contamination rates, to decreasing the fraction ρ_i of residents of community i living in rural conditions, at least as far as water and sanitation are concerned. These actions can be either targeted (e.g. implemented only in selected rural communities) or untargeted (implemented in all communities), and are obviously constrained by the available budget.

Let τ and η be the planned extent of the interventions (evaluated as the number of potentially benefited people, $\tau \leq \sum_i \rho_i K_i$) and their potential efficiency (i.e. the probability of success per effort unit). Untargeted actions can be described in the model as

$$\rho_i' = \rho_i \left(1 - \frac{\eta\tau}{\sum_j \rho_j K_j}\right),$$

where ρ_i' represents the fraction of people in community i with no access to safe water supplies and improved sanitation after action implementation.

Targeted interventions can be formulated in the model by sorting communities (according to some suitable criterion), selecting the first U ones so that $\sum_{i=1}^{U} \rho_i K_i \leq \tau$ and setting

$$\rho_i' = 1 - \eta$$

therein. Natural ranking criteria in our framework include the quantity $\rho_i \omega_i$, which quantifies transmission risk based on the rurality of living conditions and the abundance of environmental freshwater where snail populations can thrive, or the regional values of schistosomiasis prevalence. Other options (like e.g. prioritizing rural or water-rich regions, or communities with large inbound/outbound mobility fluxes) are obviously possible.

Information, Education and Communication (IEC) campaigns

The effect of IEC campaigns aimed at promoting hygiene and increasing awareness about disease transmission pathways[2] can be modeled as a decrease of the baseline exposure/contamination rates. Like in the case of WASH interventions, IEC campaigns can be either targeted or untargeted, and are subject to budget constraints.

Let τ and η be again the planned extent of the interventions ($\tau \leq \sum_i K_i$) and their supposed efficiency, respectively. Untargeted interventions can be modeled as

$$\beta_0' = \beta_0 \left(1 - \frac{\eta\tau}{\sum_i K_i}\right), \quad \chi_0' = \chi_0 \left(1 - \frac{\eta\tau}{\sum_i K_i}\right)$$

in the case of the fine-grained model set-ups M3 and M4 (similar relationships can be worked out for the coarse-grained set-ups M1 and M2).

The implementation of targeted interventions requires sorting the communities (again, according to some suitable criterion),

selecting the first U ones so that $\sum_{i=1}^{U} K_i \leq \tau$, and setting

$$\beta_0' = 1 - \eta\,, \quad \chi_0' = 1 - \eta$$

therein. The previous equations refer again to model set-ups M3 and M4, but similar relationships can be obtained for set-ups M1 and M2 as well. Like in the case of WASH interventions, ranking criteria can prioritize high-risk or high-prevalence communities, but other indicators describing access to safe water sources or sanitation facilities (available online through GAHI, Maps and data, http://www.thiswormyworld.org; last last date of access: 03/02/2017) could be used as well.

References

1. Colley, D. G., Bustinduy, A. L., Secor, W. E. & King, C. H. Human schistosomiasis. *Lancet* **383**, 2253–2264 (2014).
2. Rollinson, D. *et al.* Time to set the agenda for schistosomiasis elimination. *Acta Tropica* **128**, 423–440 (2013).
3. Ogden, S. *et al.* WASH and the neglected tropical diseases. A manual for WASH implementers. Senegal. Tech. Rep., WASH NTD. Available online at http://www.washntds.org/ (2014). Date of access: 01/08/2016.
4. Grimes, J. E. T. *et al.* The relationship between water, sanitation and schistosomiasis: a systematic review and meta-analysis. *PLoS Neglected Tropical Diseases* **8**, e3296 (2014).
5. Grimes, J. E. T. *et al.* The roles of water, sanitation and hygiene in reducing schistosomiasis: a review. *Parasites & Vectors* **8**, 156 (2015).
6. Schmidt, W.-P. The elusive effect of water and sanitation on the global burden of disease. *Tropical Medicine and International Health* **19**, 522–527 (2014).
7. Ndir, O. Situation des schistosomoses au Sénégal. In Chippaux, J. P. (ed.) *La Lutte contre les Schistosomoses en Afrique de l'Ouest*, 225–236 (IRD Editions, 2000).
8. Stensgaard, A. S. *et al.* Large-scale determinants of intestinal schistosomiasis and intermediate host snail distribution across Africa: does climate matter? *Acta Tropica* **128**, 378–390 (2013).
9. Perez-Saez, J. *et al.* A theoretical analysis of the geography of schistosomiasis in Burkina Faso highlights the roles of human mobility and water resources development in disease transmission. *PLoS Neglected Tropical Diseases* **9**, e0004127 (2015).
10. Pullan, R. L., Freeman, M. C., Gething, P. W. & Brooker, S. J. Geographical inequalities in use of improved drinking water supply and sanitation across sub-Saharan Africa: mapping and spatial analysis of cross-sectional survey data. *PLoS Medicine* **11**, e1001626 (2014).
11. Mari, L. *et al.* Modelling cholera epidemics: the role of waterways, human mobility and sanitation. *Journal of the Royal Society Interface* **9**, 376–388 (2012).
12. Wesolowski, A. *et al.* Quantifying the impact of human mobility on malaria. *Science* **6104**, 267–270 (2012).
13. Erlander, S. & Stewart, N. F. *The Gravity Model in Transportation Analysis – Theory and Extensions* (VSP Books, Zeist, The Netherlands, 1990).

14. Simini, F., González, M. C., Maritan, A. & Barabási, A. L. A universal model for mobility and migration patterns. *Nature* **484**, 96–100 (2012).

15. Palchykov, V., Mitrović, M., Jo, H. H., Saramäki, J. & Pan, R. K. Inferring human mobility using communication patterns. *Scientific Reports* **4**, 6174 (2014).

16. Wesolowski, A. *et al.* Evaluating spatial interaction models for regional mobility in sub-Saharan Africa. *PLoS Computational Biology* **11**, e1004267 (2015).

17. Gurarie, D., King, C. H. & Wang, X. A new approach to modelling schistosomiasis transmission based on stratified worm burden. *Parasitology* **137**, 1951–1965 (2010).

18. Macdonald, G. The dynamics of helminth infections, with special reference to schistosomes. *Transactions of the Royal Society of Tropical Medicine and Hygiene* **59**, 489–506 (1965).

19. Gurarie, D. & King, C. H. Population biology of *Schistosoma* mating, aggregation, and transmission breakpoints: More reliable model analysis for the end-game in communities at risk. *PLoS One* **9**, e115875 (2014).

20. Gurarie, D., King, C. H., Yoon, N. & Li, E. Refined stratified-worm-burden models that incorporate specific biological features of human and snail hosts provide better estimates of *Schistosoma* diagnosis, transmission, and control. *Parasites and Vectors* **9**, 428 (2016).

21. CIA. The World Factbook 2013–14. Tech. Rep., Central Intelligence Agency. Available online at `https://www.cia.gov/library/publications/the-world-factbook/index.html` (2014). Date of access: 01/08/2016.

22. Feng, Z., Eppert, A., Milner, F. A. & Minchella, D. J. Estimation of parameters governing the transmission dynamics of schistosomes. *Applied Mathematics Letters* **17**, 1105–1112 (2004).

23. Kheir, M. M. *et al.* Mortality due to schistosomiasis *mansoni*: a field study in Sudan. *The American Journal of Tropical Medicine and Hygiene* **60**, 307–310 (1999).

24. Lagarias, J. C., Reeds, J. A., Wright, M. H. & Wright, P. E. Convergence properties of the Nelder-Mead simplex method in low dimensions. *SIAM Journal of Optimization* **9**, 112–147 (1998).

Supplementary figures

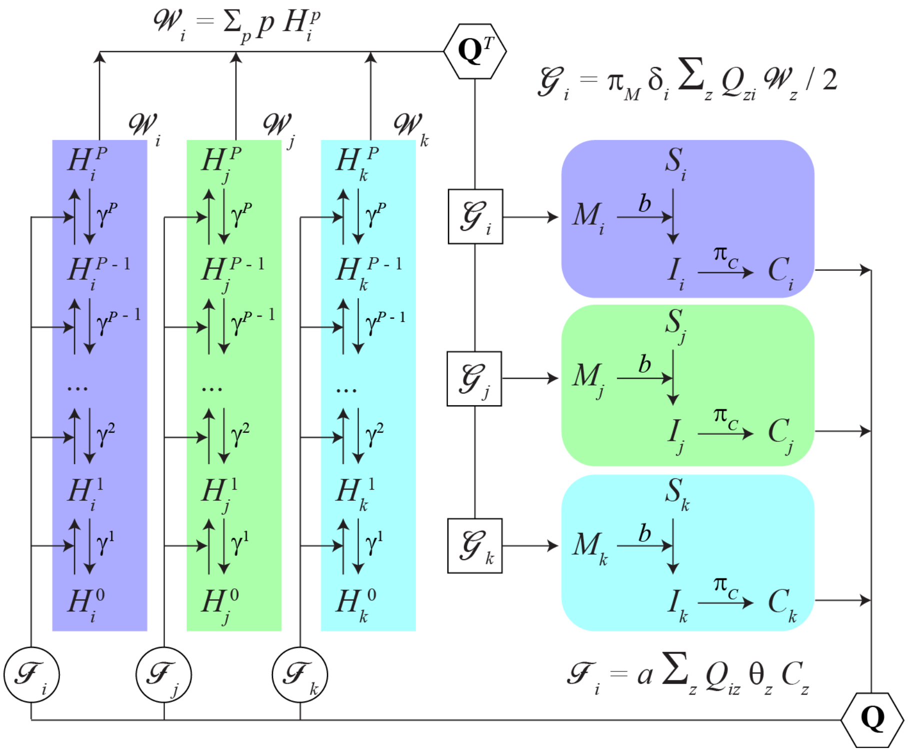

Figure S1. Schematic representation of the schistosomiasis transmission model. Rectangles represent the human components of three sample communities (say i, j and k, identified by different colors), stratified by infection class (H_i^p are hosts burdened with $0 \leq p \leq P$ parasites in community i). Rounded rectangles represent the freshwater components of the three communities, including (say, for community i) susceptible (S_i) and infected (I_i) snails, and the larval stages of the parasite (miracidia, M_i, and cercariae, C_i). Circles, squares and hexagons indicate the force of infection for the different human communities, the human contribution to freshwater infestation and mobility-related processes, respectively. See Fig. 1a in the main text for a graphical depiction of the transmission cycle of the disease, the *Methods* section in the main text for a complete description of the model and Table 1 in the main text for a description of the variables and parameters used in the model.

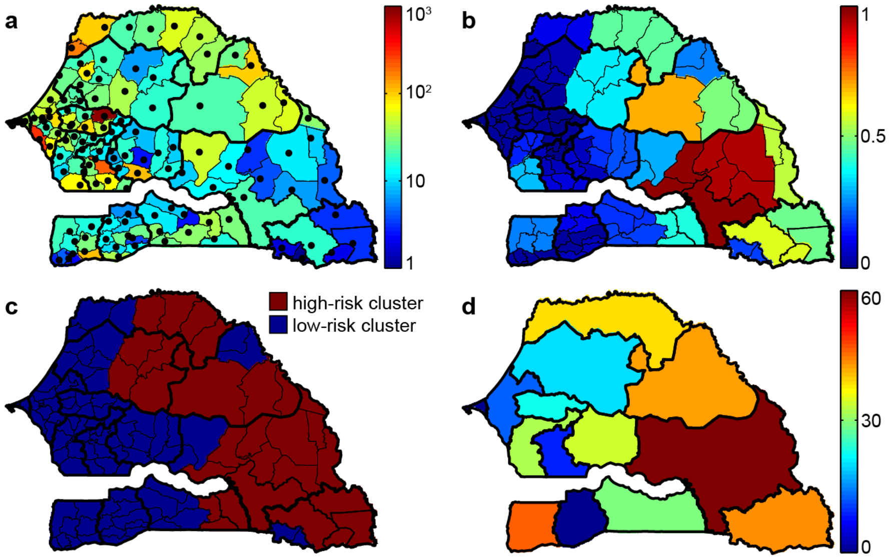

Figure S2. Data for model simulation and calibration. (**a**) Distribution of population abundance in third-level administrative units [thousands of inhabitants]. (**b**) Spatial distribution of the product between the rurality score ρ_i and the freshwater availability index ω_i (model set-ups with fine-grained environmental heterogeneity, M3 and M4). (**c**) Identification of high-risk vs. low-risk transmission communities based on the clustering of $\rho_i\omega_i$ scores (model set-ups with coarse-grained environmental heterogeneity, M1 and M2). (**d**) Regional prevalence of urogenital schistosomiasis [% of infected people] according to the current estimates of the Senegalese Ministry of Health. Maps have been created with QGIS 2.4 (QGIS Development Team, QGIS: A free and open source geographic information system, http://www.qgis.org/; last last date of access: 03/02/2017) and MATLAB R2015b (MathWorks, MATLAB, http://www.mathworks.com/products/matlab/; last last date of access: 03/02/2017).

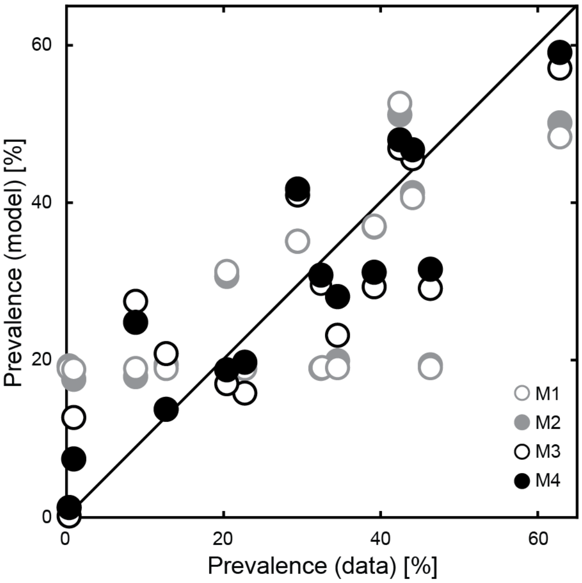

Figure S3. Calibration of the four model set-ups. Best-fit parameter values: for M1, $\beta_{low} = 4.7 \cdot 10^{-3}$ [days^{-1}], $\chi_{low} = 6.7 \cdot 10^{-4}$ [days^{-1} parasites^{-1}], $\beta_{high} = 6.8 \cdot 10^{-3}$ [days^{-1}], $\chi_{high} = 1.1 \cdot 10^{-1}$ [days^{-1} parasites^{-1}]; for M2, $\beta_{low} = 4.6 \cdot 10^{-3}$ [days^{-1}], $\chi_{low} = 8.3 \cdot 10^{-5}$ [days^{-1} parasites^{-1}], $\beta_{high} = 7.1 \cdot 10^{-3}$ [days^{-1}], $\chi_{high} = 7.3 \cdot 10^{-2}$ [days^{-1} parasites^{-1}]; for M3, $\beta_0 = 5.0 \cdot 10^{-3}$ [days^{-1}], $\chi_0 = 2.7 \cdot 10^{-3}$ [days^{-1} parasites^{-1}], $\phi = 5.2 \cdot 10^{-1}$, $\xi = 1.3 \cdot 10^{2}$; for M4, $\beta_0 = 5.5 \cdot 10^{-3}$ [days^{-1}], $\chi_0 = 2.2 \cdot 10^{-3}$ [days^{-1} parasites^{-1}], $\phi = 4.3 \cdot 10^{-1}$, $\xi = 1.1 \cdot 10^{2}$. The model set-up accounting for fine-grained spatial heterogeneity and human mobility (M4) has better overall explanatory power than the others ($R^2 = 0.76$) and is thus retained as reference model.

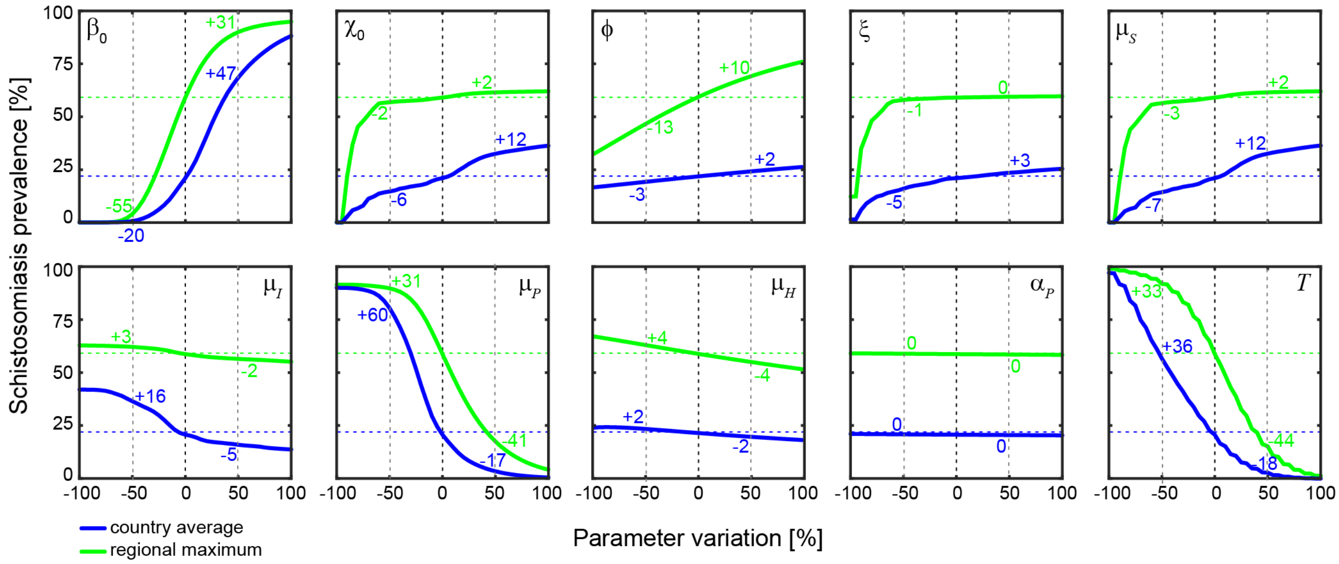

Figure S4. Sensitivity analysis of model projections with respect to parameter variations. In each panel, results are shown for simulations performed with the reference model and the best-fit parameter set (see Fig. 2 in the main text) except for the value of one parameter (label) that has instead been allowed to vary in a ±100% range with respect to its reference value. Shown are the effects of parameter variations on the country-averaged (blue) and the maximum regional (green) values of schistosomiasis prevalence. Blue/green numbers within each panel indicate deviations from the reference simulation for ±50% parameter variations.

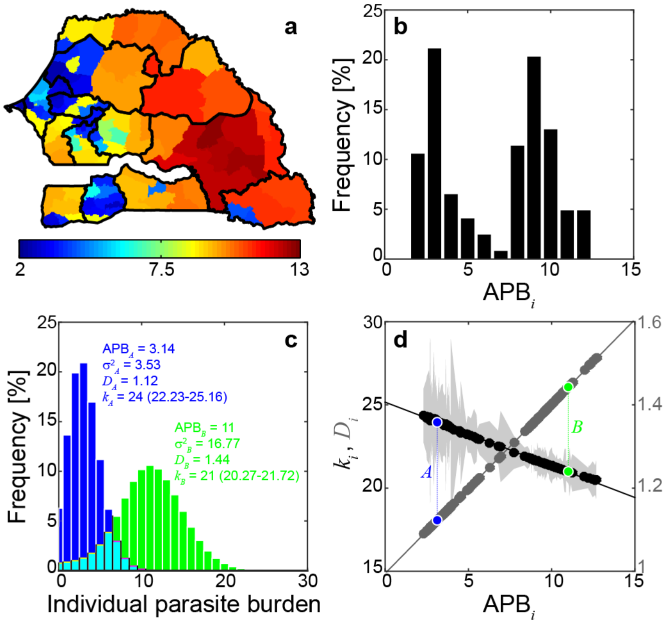

Figure S5. Within-host parasite distribution as simulated by the reference model. (**a**) Spatial distribution of the APB [number of parasites per person] in each arrondissement. (**b**) Frequency distribution of APB$_i$ (arrondissement level). (**c**) Parasite distributions within the human population of two exemplificative arrondissements. σ_i^2 indicates the sample variance of the parasite distribution within a community ($i = \{A, B\}$), $D_i = \sigma_i^2/\text{APB}_i$ is the dispersion index and k_i is the aggregation parameter of a negative binomial fitted to the simulated parasite distribution via maximum likelihood estimation (ranges are 95% confidence intervals). The values of k_i and D_i indicate relatively low parasite aggregation in both communities (aggregation being larger in the arrondissement marked as B, green, because $k_B < k_A$ and $D_B > D_A$). (**d**) Relationships between APB$_i$ and two indicators of parasite aggregation, namely the aggregation parameter k_i (black, left axis) and the dispersion index D_i (dark gray, right axis), evaluated for each arrondissement i. The area shaded in light gray is the 95% confidence interval for the estimated values of k_i. The green and the blue points indicate the arrondissements for which individual-level parasite distributions are shown in panel c. The map in panel a has been created with QGIS 2.4 (QGIS Development Team, QGIS: A free and open source geographic information system, `http://www.qgis.org/`; last last date of access: 03/02/2017) and MATLAB R2015b (MathWorks, MATLAB, `http://www.mathworks.com/products/matlab/`; last last date of access: 03/02/2017).

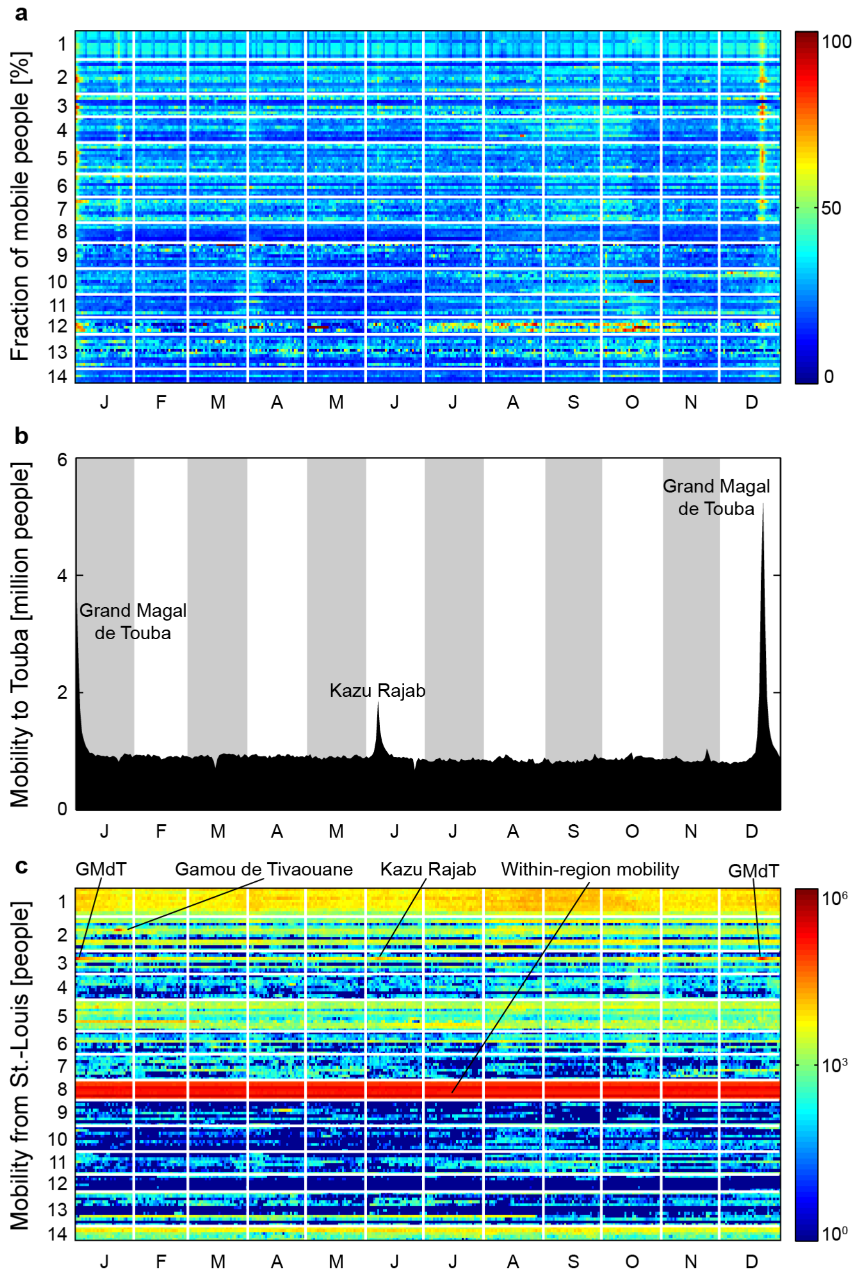

Figure S6. Examples of daily human mobility patterns in Senegal. (**a**) Overall mobility from each arrondissement $i = 1 \ldots 123$. Region labels ($r = 1 \ldots 14$) are shown for easier visual reference and are the same as in Fig. 1b in the main text. (**b**) Mobility fluxes to Touba (Ndame arrondissement, Diourbel region), evaluated as $\sum_i K_i Q_{i,\mathrm{Ndame}}$, $i = 1 \ldots 123$. Peaks of incoming mobility correspond to the most important religious gatherings held in Touba during 2013. (**c**) Mobility fluxes from Saint-Louis region (8), evaluated as $\sum_i K_i Q_{i,j}$ with $i \in$ Saint-Louis, $j = 1 \ldots 123$. Highlighted are religious gatherings that generate peaks of outgoing mobility fluxes (Grand Magal de Touba, GMdT; Gamou de Tivaouane; Kazu Rajab) and within-region mobility. Overall, remarkable mobility fluxes are also directed towards the most 'attractive' and well connected region of Dakar, or to those closest in distance to Saint-Loius (Louga, 5, and Matam, 14), which show the 'gravitational' nature of human mobility. Daily mobility patterns are defined by looking at the spatial patterns of the calls made by each user over one day (instead of one year like in Fig. 1d).
